# Evaluation of a decision aid for prenatal testing of fetal abnormalities: a cluster randomised trial [ISRCTN22532458]

**DOI:** 10.1186/1471-2458-6-96

**Published:** 2006-04-13

**Authors:** Cate Nagle, Sharon Lewis, Bettina Meiser, Sylvia Metcalfe, John B Carlin, Robin Bell, Jane Gunn, Jane Halliday

**Affiliations:** 1 Murdoch Childrens Research Institute, Flemington Road, Parkville, Victoria, 3052, Australia; 2 Department of Paediatrics, The University of Melbourne, Flemington Rd, Parkville, Victoria, 3053, Australia; 3 Department of General Practice, The University of Melbourne, Berkeley St, Carlton, Victoria, 3053, Australia; 4 School of Psychiatry, The University of New South Wales, Hospital Road, Randwick, NSW, 2052, Australia; 5 Clinical Epidemiology & Biostatistics, Royal Children's Hospital, Flemington Road, Parkville, Victoria, 3052, Australia; 6 Women's Health Program, Department of Medicine, Monash University, Central and Eastern Clinical School, Alfred Hospital, Commercial Road, Prahran, Victoria, 3181, Australia

## Abstract

**Background:**

By providing information on the relative merits and potential harms of the options available and a framework to clarify preferences, decision aids can improve knowledge and realistic expectations and decrease decisional conflict in individuals facing decisions between alternative forms of action. Decision-making about prenatal testing for fetal abnormalities is often confusing and difficult for women and the effectiveness of decision aids in this field has not been established. This study aims to test whether a decision aid for prenatal testing of fetal abnormalities, when compared to a pamphlet, improves women's informed decision-making and decreases decisional conflict.

**Methods/design:**

A cluster designed randomised controlled trial is being conducted in Victoria, Australia. Fifty General Practitioners (GPs) have been randomised to one of two arms: providing women with either a decision aid or a pamphlet. The two primary outcomes will be measured by comparing the difference in percentages of women identified as making an informed choice and the difference in mean decisional conflict scores between the two groups. Data will be collected from women using questionnaires at 14 weeks and 24 weeks gestation.

The sample size of 159 women in both arms of the trial has been calculated to detect a difference of 18% (50 to 68%) in informed choice between the two groups. The required numbers have been adjusted to accommodate the cluster design, miscarriage and participant lost – to – follow up.

Baseline characteristics of women will be summarised for both arms of the trial. Similarly, characteristics of GPs will be compared between arms.

Differences in the primary outcomes will be analysed using 'intention-to-treat' principles. Appropriate regression techniques will adjust for the effects of clustering and include covariates to adjust for the stratifying variable and major potential confounding factors.

**Discussion:**

The findings from this trial will make a significant contribution to improving women's experience of prenatal testing and will have application to a variety of maternity care settings. The evaluation of a tailored decision aid will also have implications for pregnancy care providers by identifying whether or not such a resource will support their role in providing prenatal testing information.

## Background

Peak obstetric bodies [1-3] recommend that all pregnant women, regardless of age, are provided with information on prenatal screening tests for fetal abnormality. Women often find this information difficult to understand and studies have demonstrated low levels of knowledge [4-7]. Health professionals are an important source of information for women, yet studies have demonstrated they too can also experience difficulty in understanding the inherent complexities related to screening tests [8-10]. Not surprisingly, studies have consistently demonstrated a need to improve women's capacity to make an informed decision regarding prenatal screening tests and the need for women to have a more active role in decision-making. At a minimum, women need to understand the condition(s) for which the testing is being offered, the characteristics of the test and the implications of testing [11].

Decision aids are resources that are designed to assist individuals to make specific and deliberate choices from available options by providing information on the options and outcomes relevant to the individual's health status [12]. They have been found to improve individuals' decision-making and outcomes in a variety of health related areas [13] and have been established as effective in: improving knowledge and realistic expectations of benefits and risks of options, increasing participation in decision-making and reducing decisional conflict without affecting levels of anxiety or satisfaction. Despite promising results among women of advanced maternal age using a decision aid for prenatal diagnostic testing [14], the role of decision aids for women of all ages considering prenatal screening tests has not been established.

We are undertaking a cluster randomised controlled trial to test whether a decision aid for prenatal testing of fetal abnormalities, when compared to a pamphlet, improves women's informed decision-making [11] and decreases their decisional conflict [15]. The trial, called ADEPT (A DEcision Aid for Prenatal Testing of fetal abnormalities), is being conducted in the primary health care setting, as GPs are often the first health professional a woman consults early in pregnancy. Consequently, this setting allows for provision of the allocated information at a gestation when women have the best chance to consider first and second trimester testing options.

## Methods/design

### Design of the intervention

The decision aid for prenatal testing of fetal abnormalities has been developed using the three steps of the Ottawa Decision Support framework: identifying needs, providing decision support and evaluating decision support [16].

The need for a decision aid was identified following a literature search and by conducting focus groups with the target groups relevant to the study: five focus groups with women and four with GPs were conducted in metropolitan and regional Victoria, Australia. The key finding from the GP focus groups was the difficulty GPs experienced in making screening test information appropriate, relevant and understandable to the individual women. In particular, explaining epidemiological concepts was identified as challenging. GPs experienced significant time pressures in the first consultation in pregnancy and strongly supported the development of a tailored resource. The women's focus groups revealed low level of prenatal screening knowledge and diversity in the amount of information women wanted. To accommodate this a flexible paper-based format was drafted, incorporating the use of summary tables and dot points, as well as more detailed information and a resource list. Consistently women identified difficulty with statistical and numeric expressions and this influenced the use of graphs and diagrams throughout the decision aid. Draft formats of the decision aid were piloted with women who were attending a tertiary prenatal clinic in early pregnancy.

The resultant decision aid is a 24-page information booklet and a risk report. The booklet contains information on why testing is offered, the range of chromosomal and physical abnormalities for which tests may be offered, the different types of tests available and the possible outcomes of testing. Also, within the booklet are scenarios of women's decision-making about testing, a list of other resources available and a worksheet to assist women in weighing up the benefits and risks of different options. A risk report sheet detailing an individualised risk estimation for having a pregnancy affected by Down syndrome is generated, based on an algorithm involving the woman's age and gestation [17,18]. The individual risk estimate given to women was obtained through use of a paper-based chart or a CD-ROM, and the GPs were given a choice as to which report generation process suited them best.

The decision aid is being compared to a pamphlet developed by Genetic Health Services Victoria (GHSV). The pamphlet is currently available free of charge within Victoria. It is in the form of a fold out A3 paper size and contains information on maternal age related risk, screening and diagnostic tests, a table summarising the tests available and what conditions they detect.

### Ethics

Royal Australian College of General Practitioners has granted ethics approval to conduct this trial (NREEC 03–16). The trial complies with the principles of the Helsinki Declaration [19]. A plain language statement is provided to all women (see Additional file 1) and GPs in the study and a signed consent form is obtained from all participants at the time of recruitment.

### Setting for the trial

The ADEPT study is being conducted in the primary health care setting of Victoria, one of the south-eastern states of Australia, where approximately 62,000 women give birth each year [20]. All women in Victoria booked to give birth at a publicly funded maternity setting have access to a second trimester maternal serum screening test, free of charge. Within this public system, women identified as being at 'increased risk' for fetal abnormality on the basis of their age (37 years or older) or screening test result, have access to diagnostic testing that is fully funded. Outside these criteria women incur out-of-pocket expenses for most other screening or diagnostic tests, although some costs are covered by a federally funded rebate. All serum samples are sent to a centralised laboratory at GHSV for testing.

In Victoria, the provision of information on prenatal testing options is not uniform. Some women receive information that is verbal and/or written, while others receive no information at all. The pamphlet produced by the GHSV is one of a vast array of written information used throughout the state. While the exact rate of uptake of screening tests in the state is not known, it is estimated that approximately 60% of pregnant women have some form of screening test [21] and utilisation is increasing. An abnormal screening test result was the indication for 44% of diagnostic tests in 2004, compared to 41% for maternal age alone [22].

### Cluster design

Cluster randomisation has been chosen to avoid contamination of the intervention to the control group, which would dilute the difference between the groups thus reducing the power of the study. It has been proposed that the use of cluster randomisation can be justified when the risk of contamination between groups is estimated to be greater than 30% [23]. This level of contamination could reasonably occur in the ADEPT study at the level of the GP, or by women in the intervention group sharing the decision aid with women in the control group within a variety of maternity care environments, as well as through social settings. It is also possible that the paper-based decision aid could be copied and circulated to women in the control arm of the trial.

GPs were randomised either to the intervention arm, providing women with the decision aid, or in the control arm, providing women with the GHSV pamphlet. The individual GP was used as the unit of randomisation. It was envisaged that the cluster design would have the added advantage of assisting participating GPs to adhere to the trial protocol [24], as they would be providing the same resource to all women they recruited. Data are currently being collected from questionnaires completed by women.

### Sample size

The required sample size has been adjusted for the clustered design and has been based on using a two-sided test at a 0.05 level of significance and 80% power to detect a difference of 18% (50 – 68%) in the rate of informed choice between women in the intervention and control groups. The anticipated difference in outcome measure was based on a study assessing women's knowledge of prenatal tests to detect Down Syndrome [4] and it was judged that smaller effects would not be of public health significance.

Using an intraclass correlation coefficient (ICC) of 0.05 and with 25 GPs in each arm of the trial, the design effect was calculated as 1.25. ICC within the Australian primary health context are seldom published [25], so we estimated an ICC for our sample based on an unpublished survey of 50 currently pregnant women sampled consecutively in 30 GP practices in Victoria by one of the study's investigators (JG). This adjustment resulted in a sample estimate of 159 women per arm of the trial. The sample size was further adjusted to allow for 35% attrition rate due to miscarriage in the first trimester [26] and non- return of the first questionnaire [27]. The required recruitment sample was estimated at 245 women per arm of the trial in order to provide a total of 318 respondents for analysis.

### Enrolling GPs in the ADEPT study

Geographical areas were purposively targeted to achieve a geographical spread in the location of general practices throughout the metropolitan area. As approximately one-fifth of Victorian births occur in rural areas [28], these areas were also targeted to ensure non-metropolitan representation. Regional or rural areas were identified on the basis of the number of first trimester samples sent to the centralised laboratory at GHSV in 2004.

Individual letters inviting expressions of interest in the study were sent to GPs (see Additional file 2). The contact details of GPs were obtained from professional organisations, public telephone listings and in-house databases of GPs interested in prenatal testing. Other strategies also involved advertising the ADEPT study through professional organisations and medical media.

### Selection of GPs

Eligibility criteria required that the GP estimated that s/he consults with at least 30 women in early pregnancy within a twelve-month period. Participation was limited to one GP per practice to minimise the potential for contamination previously described. In addition, it was hoped that this restriction would also increase the generalisability of the findings, as GPs who practise together are more likely to share other characteristics such as practice style and attitudes. The 50 GPs participating in the study were asked to each recruit ten women to the trial.

### Randomisation of GPs

A person independent to the study has conducted all randomisation procedures for this study. Initially, the names of the 63 GPs expressing interest in the study were randomly sorted. GPs were contacted by phone using the numerical order of the list of names, until verbal consent to be randomised to either arm was obtained from 50 GPs. This addressed the potential for selection bias inherent in cluster trials [23,24,29], where participation rates differ between the groups following allocation.

The GPs were stratified by location (metropolitan/rural) in an attempt to address the association between geographical location and women's uptake of prenatal diagnostic testing [30]. Random allocation was obtained using a computer generated random list of numbers.

### Academic detailing of GPs

Following randomisation, visits to the practices of all GPs were arranged. Prior to the visits a detailed information sheet on the ADEPT study and consent form were mailed to each GP together with a survey. The survey, collected at the visit, requested information that included a description of their role in prenatal testing and details of their usual practice including the amount of time they spend counselling women, the importance they attach to women of different age groups having adequate information and having testing. Professional history and socio-demographic details were also collected.

Members of the project team (SL & CN) conducted all visits according to an agreed practice visit schedule. The project was described in detail, as was the role of the GP and the process involved in enrolling a woman in the ADEPT study. GPs were provided with the project resources including a manual and ten information packs for women.

### Enrolling women in the ADEPT study

Each GP was asked to provide information on the ADEPT study and to offer participation to all pregnant women meeting the study's selection criteria (see Additional file 3). The risk of selection bias recognised in cluster trials [31] will be measured by way of an audit (see Additional file 4) using details collected by the GP on the age, parity and country of birth of all women meeting the selection criteria during the recruitment period. Australian GPs are primarily funded by a fee for service system and receive no funding (personal or infrastructure) for involvement in research. GPs have been reimbursed for the time spent enrolling a woman to the study ($30AUD/woman).

GPs needed to obtain written informed consent from women wanting to participate in ADEPT as well as the woman's contact details and estimated date of delivery. These details were faxed/posted to the project office. The GP provided each participating women with an information resource (decision aid or pamphlet) and the first questionnaire, as well as written instructions, an information sheet and a copy of the consent form she signed.

### Inclusion criteria for women

Pregnant women attending a participating GP were eligible to participate provided they were aged 18 years or older and were equal to, or less than, 12 weeks gestation.

### Exclusion criteria for women

Women were excluded if they were non-English speaking, were unable to give written informed consent or required genetic counselling due to a family history of an inherited condition or recurrent risk for fetal abnormality. Exclusion criteria also included having already undertaken testing for fetal abnormality in this pregnancy, experiencing vaginal bleeding or currently having a known multiple pregnancy.

### Data collection from women

Women were asked to complete two questionnaires, the first by 14 weeks gestation and the second at approximately 24 weeks gestation. The initial questionnaire was given to the woman by her GP and the second questionnaire was forwarded to her postal address by the project team. Stamped addressed envelopes have been provided for the return of both questionnaires.

Once written informed consent was obtained from the woman by her GP, an enrolment form detailing the date, woman's name, contact details and estimated due date was faxed to the study office. This information was entered into a database to manage the reminder system for the questionnaires as well as the mail-out of the second questionnaire.

Using a modification of the technique described by Dillman [32], a two-stage reminder system is in place to maximise the response rate of the questionnaires. Women are contacted with a reminder letter two weeks after the questionnaire is due if it has not been received. A phone call reminder follows in a further two weeks if the questionnaire remains unreturned.

### Blinding

Blinding of researchers was not practical in this study, however a person independent of the study has kept the master list of all randomised groups and will audit the integrity of the randomisation prior to analysis.

Participating women were made aware that two methods of providing information were being trialed but were not advised of the alternative resource to the one they received.

### Allocation concealment

Concealment of allocation was achieved by obtaining GP's verbal consent to be randomised prior to the conduct of the randomisation. The research team members were not advised of the allocation until consent had been obtained from all GPs. GPs were unaware of the arm of the trial to which they had been randomised until the time of the practice visit.

### Data entry quality control

Prior to analysis, a random sample of ten percent of the data entry will be audited. A person independent of the study will conduct the sampling and audit. The rate of accuracy will be reported.

### Scales

ADEPT is using validated and psychometrically robust self-report scales.

The Multi-dimensional Measure of Informed Choice (MMIC) [11] scale defines an informed choice as one that is based on relevant knowledge, is consistent with a person's values and is behaviourally implemented. Three dimensions are incorporated in this measure: knowledge, attitude and uptake. There are eight knowledge items and four items on attitudes to screening tests. A record of test uptake is included in the measure. Dichotomous outcomes are classified as informed choice or uninformed choice. The classification of an informed choice is determined by a score higher than the midpoint of the knowledge scale (>4), higher than the midpoint of the attitude scale (>12) and having the test. Equally, a choice is classified as informed when a score is higher than the midpoint of the knowledge scale (>4), less than the midpoint of the attitude scale (equal or <12) and not having the test [33].

The Decisional Conflict Scale [15] contains 16 items divided into three sub-scales: uncertainty about selection of alternatives; specific factors contributing to uncertainty and perceived effectiveness of decision-making. Respondents use a five-point Likert scale (1 = strongly agree to 5 = strongly disagree). The mean scores are reported for the total scale and each sub-scale. Higher scores indicate higher decisional conflict.

The Attitudes to the Fetus/Neonate [34] measure consists of 15 adjectives and respondents use a seven point scale (0 = Not at all to 7 = extremely) to describe their feelings in relation to being pregnant (nine items) and towards the fetus (six items). By reversing specific items the mean scores are calculated for attitudes to pregnancy and the fetus, with higher scores indicating a more positive attitude.

The Edinburgh Postnatal Depression Scale [35] has been validated to use prenatally. It contains ten items and respondents have four options available to indicate the frequency of the event based on their experience of the previous week. With the reversing of specific responses, items are scored (1–4) and women can be classified as probably clinically depressed (≥ 13) or not (<13).

The short version of the Speilberger State- Trait Anxiety Inventory (STAI – State) contains six items [36]. Respondents use a four-point Likert scale to indicate how they feel now (1 = Not at all to 4 = Very much). With the reversing of specific responses, mean scores are reported with higher scores indicating greater anxiety.

The Satisfaction with Decision [37] scale contains five items and respondents use a five point Likert scale (1 = strongly agree to 5 = strongly disagree). With the reversing of specific responses, mean scores are calculated with higher scores indicating greater satisfaction.

### Outcomes

The two primary outcomes for the study are informed choice and decisional conflict. Informed choice will be measured by the percentage of women in each arm of the trial identified as making an informed choice using the MMIC [11]. Decisional conflict will be measured by the difference in mean scores of the Decisional Conflict Scale [15] between women in each arm. Both primary outcomes will be measured based on data from the first questionnaire to reflect women's experience closest to the time they were making decisions about prenatal testing.

Secondary outcomes include attitudes to the pregnancy/fetus, depression, anxiety, and acceptability of the written information by women and GPs. These outcomes will be measured using data from the first questionnaire. Women's satisfaction with their decision [37] will be assessed using data from the second questionnaire.

Changes in primary and secondary outcomes will be measured by comparing responses from the second questionnaire with the first. Attitudes to the pregnancy/fetus will be assessed using the scale developed by Reading et al (1984) [34] by measuring the difference in mean scores between women in both arms of the trial. Depression will be measured using the difference in percentages of women in both arms of the trial identified as being probably clinically depressed on the EPDS [35]. Anxiety will be measured as the difference in the mean Anxiety STAI-State (short version) score of women in both arms of the trial [36]. The acceptability of the decision aid/pamphlet to women and GPs will be measured by comparing the difference in proportions of descriptive items between women in both arms of the trial and comparing content analysis of open-ended comments.

Satisfaction with decision making will be measured using the difference in mean Satisfaction with Decision Scale [37] scores between women in both arms of the trial.

## Discussion

Standard guidelines for statistical reporting of clinical trials will be followed [38] with additional information relating to the cluster design [31,39,40]. Baseline characteristics of women including age group, marital status, educational level, income source, country of birth, religion will be compared between the intervention and control arms of the trial. In addition, obstetric history, previous experience with prenatal screening tests, perception of risk of Down syndrome and personal experience with individuals who have disabilities such as Down syndrome will be compared between arms of the trial. GPs will be compared between arms in relation to age, gender, country of graduation, years of general practice, postgraduate education and personal and professional experience with individuals who have disabilities like Down syndrome. In addition we will compare GPs in both arms of the trial on the basis of the average time spent informing women of prenatal tests and the importance they attach both to providing this information and to women having prenatal testing.

Differences in the primary outcomes will be analysed using 'intention-to-treat' principles. The primary outcome of informed choice is binary and analysis will use logistic regression to evaluate the difference in proportions between the two arms of the trial. Linear regression will be used for the other primary outcome, decisional conflict, to evaluate the difference in mean scores between the two arms of the trial. Adjustment will be made for the stratification variable, geographical location (metropolitan/rural), and the following major potential confounding factors: maternal age, education level, gravidity, socioeconomic status and country of birth. Confidence intervals and P values will be adjusted for the effect of clustering and loss to follow-up within clusters will be reported.

Secondary outcomes will use appropriate regression techniques and covariate adjustments.

## Competing interests

The author(s) declare they have no completing interests.

## Authors' contributions

JH generated the original research question and together with JG, RB, SM, BM & JC developed the project and obtained funding. This group together with SL & CN refined the protocol. CN & SL developed the materials used in the trial with input from other authors. CN wrote the first draft of this paper and all other authors contributed to subsequent drafts and approved the final version.

## Pre-publication history

The pre-publication history for this paper can be accessed here:



## Supplementary Material

Additional file 1 Woman's information statement and consent form. This file contains the information provided to women and the consent form women signed for the ADEPT study. Click here for file

Additional file 2 Letter of invitation sent to General Practitioners. This file contains the content of the letter of invitation to participate in the ADEPT study sent to all GPs in the targeted geographical areas Click here for file

Additional file 3 Flow-chart of enrolling a woman in the ADEPT study. This file contains details of the selection criteria and a flow diagram of the process of enrolment. Click here for file

Additional file 4 Audit of recruitment profile. This file contains the audit form used by GPs to collect demographic data on all women eligible to participate in the trial. Click here for file
