# Supplementary material for: Evaluation of a decision aid for prenatal testing of fetal abnormalities: a cluster randomised trial [ISRCTN22532458]
Source: BMC Public Health. 2006 Apr 13;6:96. doi: 10.1186/1471-2458-6-96 (PMC1479329; doi:10.1186/1471-2458-6-96)
Supplement: Additional file 1 — Woman's information statement and consent form. This file contains the information provided to women and the consent form women signed for the ADEPT study. [file 1471-2458-6-96-S1.doc]

Additional file 1 WOMAN’S INFORMATION STATEMENT AND CONSENT FORM

Project No: 03-16 Version 2 Date 22/01/04

Title of Project: Evaluating a Decision Aid for Prenatal Testing for Fetal Abnormality (ADEPT)

Principal Researchers: Jane Halliday, Jane Gunn, Robin Bell, Sharon Lewis and Cate Nagle

Thank you for taking the time to read this Information Statement.

This information statement is printed double sided on one (1) page. The consent form is attached on a separate page. Please make sure you have all the pages.

###### Your consent

You are invited to participate in a research project called ADEPT. The purpose of this information statement is to explain to you clearly and openly all the steps and procedures of this project. The information is to help you to decide whether or not you would like to take part in the research.

Please read this information carefully. You can ask questions about anything in it. You may also wish to talk about the project with your partner, friends or family. When you understand what the project is about, you can sign the consent form attached if you wish to take part. You will be given a copy of this information and the consent form to keep.

###### What is ADEPT about?

Early in pregnancy women receive information on and the offer of testing for fetal abnormalities such as Down syndrome. We know this information can be confusing and is often difficult for women to understand. At present we do not know the best way to present the information to women. In order to improve the information women receive, we are comparing two different types of information to find out which type women find most helpful in making these decisions.

We are hoping you will agree to participate. Whether or not you are thinking of testing for fetal abnormality and whether or not you have decided on testing, your feedback will be very useful to us. You have been invited to take part, as your GP is one of the 50 Victorian GPs in the study. We anticipate each GP in the project will enrol 10 women interested in participating. GPs will be reimbursed thirty dollars per participant for their time in supporting the study.

If you agree to take part in ADEPT, your GP will give you one of two different types of information on testing for fetal abnormality to take home and read. We do not know what type women find most useful. The information you receive may be additional to the care and information your GP usually provides Your GP has been randomly allocated (like tossing a coin) to one of two groups giving women one type of information or the other. Neither you nor your GP are able to choose which one you get. This is an important part of the study so we can evaluate the different resources in a meaningful way.

###### What will I need to do?

Take the information on prenatal testing given to you by your GP home and read it.

After reading the information and after making your decision about testing please complete Questionnaire 1 (takes 15 minutes) and post it back to us.

If you haven’t made a decision about testing by 14 weeks of pregnancy please complete Questionnaire 1 at this time.

We will send you two (2) more questionnaires, one at 6 months of pregnancy and a final questionnaire about three (3) months after your baby is due. Each questionnaire will take about 10 minutes to complete.

We will need you to provide the ADEPT team with your contact details for this purpose.

###### Is there likely to be a benefit to me?

We anticipate that most women participating in the project will learn new information about prenatal testing for fetal abnormalities.

Is there likely to a benefit to other people in the future?

As a result of this study we will be able to identify which type of information is most helpful for women considering testing for fetal abnormality in pregnancy. This will be valuable information to pregnant women throughout Australia.

What are the possible risks and/or side effects?

It is recommended that all pregnant women receive information on and the offer of testing for fetal abnormality, so it is unlikely that any additional risks will result by participating in the ADEPT study. However if this information causes questions or concerns for any woman, contact numbers of genetic counselling services are provided.

###### What will be done to make sure the information is confidential?

Your questionnaires will have a code number on them, not your name, to protect your privacy. The information you provide will be treated as confidential and only members of the project team will have access. The information you provide will only be used to answer the important questions of this project and will be kept confidential subject to legal limitations. All information provided will be kept secure, in locked storage at the Murdoch Childrens Research Institute.

Your participation in completing this questionnaire is **voluntary** and you may withdraw at any time without explanation and with no effect on your care. To withdraw during the study you may return the questionnaire unanswered or contact Cate Nagle on 03 8341 6370

If you do not contact us, we will assume you are happy to receive the questionnaires.

Any information published from this study will use grouped data so no individual will be identified. A summary of the final results will be available early in 2006 and may be requested by phone or by completing the section on Questionnaire 3.

The ADEPT project follows the guidelines of the National Statement on Ethical Conduct in Research Involving Humans (1999).

The ADEPT project has been approved by the Royal Australian College of General Practitioners

National Research & Evaluation Ethics Committee (03-16)

| If you would like more information about the study or if you need to contact a study representative, please call :  Name: Cate Nagle Contact telephone: 03 8341 6370 or 0417 853574  If you have any concerns about the study, and would like to speak to someone independent of the study, please contact Catherine Lees, Executive Officer, Royal Australian College of General Practitioners NREEC Telephone 03 8699 0481 |
| --- |

PARTICIPANT INFORMED CONSENT FORM

| Study ID No |  |  |  |  |
| --- | --- | --- | --- | --- |

Version 2 Dated 22/01/04

| Title of Project: Evaluating a Decision Aid for Prenatal Testing (ADEPT) | |
| --- | --- |
| Principal Investigator(s) | Researchers: Jane Halliday, Jane Gunn, Robin Bell, Sharon Lewis and Cate Nagle |

| I, | |  |
| --- | --- | --- |
| voluntarily consent to taking part in this research project, which has been explained to me by | | |
| Dr |  | |
|  | | |
| I have received a Participant Information Statement (version 2) to keep and I believe I understand the purpose, extent and possible effects of my involvement.  I have been asked if I would like to have a family member or friend with me while the project was explained.  I have had an opportunity to ask questions and I am satisfied with the answers I have received.  I understand that the researcher has agreed not to reveal results of any information involving me, subject to legal requirements.  If information about this project is published or presented in any public form, I understand that the researcher will not reveal my identity.  I understand that if I refuse to consent, or if I withdraw from the study at any time without explanation, this will not affect my access to the best available treatment options and care.  I understand I will receive a copy of this consent form. | | |

| SIGNATURE |  | Date |  |
| --- | --- | --- | --- |

| I have explained the study to the participant who has signed above, and believe that they understand the purpose, extent and possible effects of their involvement in this study. | | | |
| --- | --- | --- | --- |
| GP’s SIGNATURE |  | Date |  |
