# Supplementary material for: Evaluation of a decision aid for prenatal testing of fetal abnormalities: a cluster randomised trial [ISRCTN22532458]
Source: BMC Public Health. 2006 Apr 13;6:96. doi: 10.1186/1471-2458-6-96 (PMC1479329; doi:10.1186/1471-2458-6-96)
Supplement: Additional file 3 — Flow-chart of enrolling a woman in the ADEPT study. This file contains details of the selection criteria and a flow diagram of the process of enrolment. [file 1471-2458-6-96-S3.doc]

Additional file 3 Flowchart of enrolling a woman in the ADEPT study

Woman presents

in

early pregnancy

A woman is ELIGIBLE if she is:

Aged 18yrs or older

≤ 12 weeks gestation

Can read and write in English

Provide usual care

YES

Eligible for ADEPT

NO

Not eligible for ADEPT

No further action

**Offer** ADEPT

Woman to receive information material

Woman to complete 2 surveys in pregnancy and one after birth.

YES to

ADEPT

Complete recruitment status audit on back of the wheel

Complete recruitment status audit on back of the wheel

Complete the woman’s contact details (Yellow page)

Obtain signed consent (Blue page)

Fax/post consent and contact details to MCRI

Provide Information pack to the woman

No to

ADEPT

A woman is NOT ELIGIBLE if she:

has a known multiple pregnancy

has current vaginal bleeding

has had a previous pregnancy affected by a chromosomal abnormality

is unable to give informed consent

has already had testing this pregnancy

Consider each woman’s eligibility for ADEPT
