# Supplementary material for: Evaluation of a decision aid for prenatal testing of fetal abnormalities: a cluster randomised trial [ISRCTN22532458]
Source: BMC Public Health. 2006 Apr 13;6:96. doi: 10.1186/1471-2458-6-96 (PMC1479329; doi:10.1186/1471-2458-6-96)
Supplement: Supplementary file 3 [file 1471-2458-6-96-S4.doc]

Additional file 4: AUDIT of recruitment profile

ADEPT Office: 03 8341 6370

GP Study No ☐ ☐ ☐ ☐

Please complete these details for **all women eligible for ADEPT**
(ie 12 weeks gestation or less, English speaking) until **10** women are recruited.

Number of women	Date	Woman's initials	Age	Country of birth	Parity	Recruitment Status (Y/N)	If not recruited please document reasons (Codes below)
1							
2							
3							
4							
5							
6							
7							
8							
9							
10							
11							
12							
13							
14							
15							
16							
17							
18							
19							
20							

Codes for women not participating:
1 = Not interested in screening information; 2 = Not interested in taking part in the study; 3 = woman's time constraints; 4 = GP's time constraints; 5 = GP forgot to ask; 6 = Other (please specify)
