# Supplementary material for: Evaluation of a decision aid for prenatal testing of fetal abnormalities: a cluster randomised trial [ISRCTN22532458]
Source: BMC Public Health. 2006 Apr 13;6:96. doi: 10.1186/1471-2458-6-96 (PMC1479329; doi:10.1186/1471-2458-6-96)
Supplement: Additional file 2 — Letter of invitation sent to General Practitioners. This file contains the content of the letter of invitation to participate in the ADEPT study sent to all GPs in the targeted geographical areas [file 1471-2458-6-96-S2.doc]

Additional file 2	Letter of invitation sent to General Practitioners

Dear Dr << >>

Do you often discuss prenatal testing for fetal abnormalities with women?

Are you interested in trialing an information resource with women in early pregnancy?

Do you see at least 30 women a year to confirm pregnancy?

If YES, read on…….

From June 2004, we are enrolling 50 Victorian GPs in a randomised controlled trial where GPs will be randomly allocated to receive one of two types of information to give pregnant women.

GPs will be required to recruit 10 consecutive women in early pregnancy, obtain their informed consent and collect brief details on any woman who declines participation. You will be well resourced and supported during the recruitment period and we will schedule a practice visit at your convenience. The recruitment process is streamlined to minimise inconvenience. GPs will be remunerated for their time at $30 / woman recruited.

Women will receive the allocated resource package and be asked to complete three questionnaires: at 14 weeks; at 24 weeks and three months post-partum. We will co-ordinate obtaining the information from women on the relative impact the resources have on supporting women’s decision making.

Make the most of this unique opportunity. Only one GP per practice is able to participate. If you are unable to take part, please circulate this letter to your colleagues. To express your interest in participating, please complete the section below and return it to the MRCI.

Yours Sincerely

Cate Nagle

(On behalf of the ADEPT team: Jane Halliday, Jane Gunn, Robin Bell, Sharon Lewis, Sylvia Metcalfe, Bettina Meiser and John Carlin)

Please fax the following details to 03 9348 1391 or mail to Cate Nagle at Murdoch Childrens Research Institute, Public Health Genetics Unit, 10^th^ Floor , Royal Children’s Hospital, Flemington Rd., Parkville 3053

| Name: | |
| --- | --- |
| Practice name and address: | |
| Phone number: | Fax Number: |
| Email address: | |
| What computer software does your practice use for patient care/records? | |
